# On Diseases of the Hip-Joint; with Some Observations on Deformities of the Chest

**Published:** 1836-07

**Authors:** 


Art. XVII.—*On Diseases of the Hip-joint, with some Observations on Deformities of the Chest.* By William Coulson, Member of the Royal College of Surgeons, &c.—*London,* 1836. 8vo. pp. 32; with a Plate.

This is the reprint of a Lecture published some time ago in the Medical Gazette; and, in noticing it, we must express our surprise that a surgeon of the well-known talent and experience of Mr. Coulson should have consented to its reappearance in a distinct form. It is an extremely good lecture to be delivered to students, and it formed a very appropriate communication to the respectable journal in which it first appeared; but, assuredly its contents do not entitle it to an independent existence; nor will it add, in any degree, to the well-established reputation of its author. It, in fact, contains nothing new, or nothing that is old put in a new light. At the same time, we may add that if any student, unacquainted with the subject, wishes to obtain good general notions of the nature and proper mode of treating the hip-joint disease, he will be gratified by the perusal of Mr. Coulson's pamphlet, and the inspection of the neat little plate (from Rust's *Arthrokakologie,*) which accompanies it.

---

